# Association Study Reveals Genetic Loci Responsible for Arsenic, Cadmium and Lead Accumulation in Rice Grain in Contaminated Farmlands

**DOI:** 10.3389/fpls.2019.00061

**Published:** 2019-02-05

**Authors:** Xiuyan Liu, Sunlu Chen, Mingxue Chen, Guangyong Zheng, Yu Peng, Xiaoliang Shi, Ping Qin, Xiangyang Xu, Sheng Teng

**Affiliations:** ^1^College of Materials and Environmental Engineering, Hangzhou Dianzi University, Hangzhou, China; ^2^Laboratory of Photosynthesis and Environmental Biology, CAS Center for Excellence in Molecular Plant Sciences, Shanghai Institute of Plant Physiology and Ecology, Chinese Academy of Sciences, Shanghai, China; ^3^Key Laboratory for Water Pollution Control and Environmental Safety of Zhejiang Province, Zhejiang University, Hangzhou, China; ^4^State Key Laboratory of Crop Genetics and Germplasm Enhancement, College of Agriculture, Nanjing Agricultural University, Nanjing, China; ^5^China National Rice Research Institute, Hangzhou, China; ^6^Bio-Med Big Data Center, CAS Key Laboratory of Computational Biology, CAS-MPG Partner Institute for Computational Biology, Shanghai Institutes for Biological Sciences, Chinese Academy of Sciences, Shanghai, China

**Keywords:** heavy metal, contamination, grain, association mapping, variation, transporter, *Oryza sativa*

## Abstract

Accumulation of toxic heavy metals and metalloids (THMMs) in crop grain remarkably affects food safety and human health. Reducing the content of THMMs in grain requires the identification and manipulation of the genes regulating their accumulation. This study aimed to determine the genetic variations affecting grain THMM accumulation in rice by using association mapping. We used 276 accessions with 416 K single nucleotide polymorphisms (SNPs) and performed genome-wide association analysis of grain THMM concentrations in rice grown in heavily multi-contaminated farmlands. We detected 22, 17, and 21 quantitative trait loci (QTLs) for grain arsenic, cadmium, and lead concentrations, respectively. Both inter- and intra-subpopulation variants accounted for these QTLs. Most QTLs contained no known THMM-related genes and represented unidentified novel genes. We examined the candidate genes in *qGAS1*, a QTL for grain arsenic concentration with the best *P*-value detected for the entire population. We speculated that a transport protein of the multidrug and toxin extrusion family could be the candidate gene for this QTL. Our study suggested that the genetic regulation of grain THMM accumulation is very complex and largely unknown. The QTLs and SNPs identified in this study might help in the identification of new genes regulating THMM accumulation and aid in marker-assisted breeding of rice with low grain THMM content.

## Introduction

Farmlands, especially those near mines, are subjected to THMM contamination (Loska et al., 2004; Wei and Yang, 2010; Li et al., 2014). THMMs in the soil, such as arsenic (As), cadmium (Cd), and lead (Pb), are absorbed and accumulated in crop plants, which poses a serious risk for food safety (Clemens and Ma, 2016). Rice (*Oryza sativa* L.) is a major food crop consumed by more than one-half of the world population. When rice is grown in contaminated farmlands or irrigated with contaminated water, high levels of THMMs accumulate in grain and adversely affect human health (Clemens and Ma, 2016). In Japan, consumption of Cd-contaminated rice caused the Itai–itai disease (Yamagata and Shigematsu, 1970; Horiguchi et al., 1994). In Bangladesh, China, and India, the elevation of As content in rice significantly increased the risk of cancer (Meharg et al., 2009; Melkonian et al., 2013). In China, grain enriched with Pb concentrations were detected in field-collected brown rice and market-collected white rice from mine-impacted and E-waste recycling areas (Fangmin et al., 2006; Fu et al., 2008; Williams et al., 2009; Norton et al., 2014b). Pb toxicity can cause gastrointestinal disturbances, renal damage, and neurological effects and adversely affect the intellectual ability of children (Canfield et al., 2003; Fewtrell et al., 2003).

In plants, the accumulation abilities of THMMs are quantitative traits, and their transport and metabolism are regulated via complex processes (Clemens and Ma, 2016). Identification and manipulation of THMM-related genes could help reduce the THMM content in crops. Arsenite, the primary form of As in paddy fields, is absorbed by aquaporins (water channels) such as OsLsi1 in rice roots and translocated to the xylem by OsLsi2 (Bienert et al., 2008; Ma et al., 2008). Mutations in OsLsi1 decrease arsenite uptake, and those in OsLsi2 remarkably reduce As accumulation in the shoots and grain (Ma et al., 2008). The overexpression of two other aquaporins OsNIP1;1 and OsNIP3;3 can reduce grain arsenite accumulation by disrupting its radial transport in root (Sun et al., 2018). OsLsi2 also functions in arsenite storage in rice nodes, which significantly affects As distribution to the grain (Chen et al., 2015). In the node, arsenite is mainly chelated by phytochelatins, and the complexes are then transported into the vacuole by OsABCC1, which reduces grain As accumulation (Song et al., 2014; Chen et al., 2015). In rice, the uptake of arsenate, another form of As, is mediated by phosphate transporters such as OsPT1, and their disruption decreases arsenate uptake and As accumulation (Kamiya et al., 2013; Wang et al., 2016; Cao et al., 2017; Ye et al., 2017). In cells, arsenate is reduced to arsenite by arsenate reductases (Chao et al., 2014; Shi et al., 2016; Xu et al., 2017). The overexpression of rice arsenate reductases OsHAC1;1, OsHAC1;2, or OsHAC4 decreases grain As accumulation (Shi et al., 2016; Xu et al., 2017).

The uptake of Cd is mediated by OsNramp5 in rice roots; Cd can be transported into the vacuole by OsHMA3 (Ueno et al., 2010; Miyadate et al., 2011; Sasaki et al., 2012, 2014). The overexpression of OsHMA3 reduces grain Cd accumulation (Ueno et al., 2010). OsHMA2 can transport Cd from the apoplast to the symplast to facilitate its translocation via the phloem (Satoh-Nagasawa et al., 2012; Takahashi et al., 2012; Yamaji et al., 2013). OsLCT1, a low-affinity cation transporter, also regulates Cd transport into rice grain, and its knockdown impairs grain Cd accumulation (Uraguchi et al., 2011). Further, transporters located in the rice nodes have been shown to control Cd storage in plants, thereby impacting the regulation of Cd distribution in developing grain (Huang et al., 2017). Recently, a defensin-like protein (CAL1) was found to chelate Cd and facilitate its secretion into extracellular spaces (Luo et al., 2018). However, the genes responsible for Pb transport and metabolism in rice are not yet known (Clemens and Ma, 2016).

Genome-wide association study by using natural populations is a powerful and efficient strategy to detect genetic variations and alleles of various phenotypes and agronomic traits (Huang and Han, 2014; Zhang et al., 2016). Variations affecting grain THMM accumulation in rice have also been investigated using association mapping. Norton et al. (2014a) completed the association mapping of grain As concentration in rice using 300 accessions and 37 K SNPs. Very recently, Yang et al. (2018) reported the association mapping of ionomic variations of rice, including As, Cd, and Pb concentrations in grain. However, none of these studies was performed in heavily multi-contaminated soil, and such contamination is known to occur in fields, especially those located near mines. In this study, we used a rice population of 276 accessions with 416 K SNPs to complete the association mapping of grain THMM (As, Cd, and Pb) concentrations in rice grown in heavily multi-contaminated farmlands. We successfully identified 60 quantitative trait loci (QTLs; 55 non-redundant loci) for grain THMM concentrations. The majority of these loci were previously unidentified. We successfully predicted the possible candidate gene for a QTL with the best *P*-value in the entire population.

## Materials and Methods

### Rice Population and Genotyping

The rice population was from a global-wide rice diversity panel consisting of 413 inbred accessions (Zhao et al., 2011). In all, 276 accessions with suitable heading date time and good germination performance were selected for field experiments (Supplementary Table S1). These accessions were previously genotyped using three types of SNP arrays (1536, 44 K, and 700 K SNP chips) (Zhao et al., 2010, 2011; McCouch et al., 2016). We successfully extracted 415 754 high-quality SNPs (missing rate, <0.2; minor allele frequency, >0.05) from these array data for the 276 accessions. About 45% of the SNPs were located within genes, half of which were distributed within exons; the remaining 55% of SNPs were located in intergenic regions, half of which were distributed in putative regulatory regions (2 kb before a transcriptional start site) (McCouch et al., 2016). We also extracted high-quality SNPs for each investigated subpopulation (190,616, 198,790, 117,880, and 147,039 SNPs, respectively). The SNP data were processed using PLINK version 1.07 (Purcell et al., 2007). Based on these 416 K SNPs, we conducted PC analysis by using GCTA version 1.91 (Yang et al., 2011). Population structure was analyzed using fastSTRUCTURE, and the optimum subpopulation number was determined to be *K* = 5 (Raj et al., 2014; McCouch et al., 2016).

### Field Experiments in Contaminated Farmlands

The rice plants were cultivated in farms, which were reported to be heavily contaminated by mining tailings from nearby abandoned mines (Wang et al., 2008; Wu et al., 2013) in Shangyu, Shaoxing City, Zhejiang Province, China (N30°00′14′′, E120°46′39′′) in 2014 and 2015. The soil levels of As, Cd, and Pb were about 3,000, 4.0, and 15,000 mg/kg, respectively, which are considerably higher than the environmental quality standard for soils in China, as well as soil background levels of THMMs in Zhejiang Province (Wang et al., 2008; Wu et al., 2013). For each year, each accession was arranged in a randomized complete block design into two-row plots with 12 plants per row at spacing of 20 cm × 17 cm with three replications. The farms were managed under flooded condition. Seeds for each accession were bulk-harvested from the eight central plants from each replication at the maturing stage. Before the grain THMM concentrations were determined, the seeds were dried for 7 days and then stored for 3 months.

### Evaluation of Grain THMM Concentrations

The de-hulled seeds (brown rice) of each accession for each year were milled into fine flour, followed by filtering (0.180 mm). For each sample, 0.25 g filtered powder was placed into a 50 ml polypropylene digestion vessel containing 8 ml nitric acid, and then mixed in a graphite furnace (Agilent Technologies, CA) at 65°C for 30 min. The mixture was digested at 120°C until 3 ml was left. After cooling, 3 ml hydrogen peroxide was added to each digestion vessel. The mixture was again digested until 1 ml was left. After digestion, the solution was diluted to 50 ml with ultrapure water and mixed to uniformity. The diluted solutions were analyzed for As, Cd, and Pb by using inductively coupled plasma mass spectroscopy (Agilent 7500A; Agilent Technologies, CA). The rice reference material (GBW10045) was used as the standard. Three technological repetitions were performed for each sample, and three biological repetitions were used for each accession. The averages of 2-year repeats were used for data analyses. Correlation tests showed a significant correlation between each pair of 2-year repeats (*P* = 4.52 × 10^-4^ to 2.63 × 10^-53^; Supplementary Table S2). Two-way ANOVA was employed to examine the genotype and year effects on grain THMM concentrations (Supplementary Table S3).

### Genome-Wide Association Study

We examined the LD decay for the 276 lines by using 416 K SNPs and PopLDdecay version 3.4 (Zhang et al., 2018). The following parameters were used: maximum distance between two SNPs, 500 kb; missing rate, <0.2; minor allele frequency, >0.05. We also calculated LD decays for each subpopulation. The widely accepted linear mixed model was used for genome-wide association study and was implemented in GEMMA version 0.97 (Zhou and Stephens, 2012). The population structure was controlled using a relatedness matrix that was calculated using all high-quality SNPs mentioned above. In addition to association mapping for the entire population, we performed association mapping for the respective subpopulations (not including the admixed subpopulation and the very limited aromatic subpopulation). For association mapping for subpopulations, high-quality SNP datasets were obtained for each subpopulation as mentioned above, and a corresponding relatedness matrix was constructed using the respective SNP dataset. Null hypothesis of no association was tested, and the statistical probability values were calculated using Wald test in GEMMA. The threshold for significance was determined according to a previous study (McCouch et al., 2016). In the case of a strong background in some mapping results (for grain Cd and Pb concentrations in the *AUS* subpopulation), we adjusted the threshold for significance by using Bonferroni correction for multiple comparisons at a false discovery rate of 0.05 (*P*-value = 0.05/SNP number).

### Candidate Gene Analyses

Considering the balance between sensitivity and reliability, the potential candidate genes were predicted within a 400 kb genomic region for each locus (±200 kb of the peak SNPs). For the loci with multiple significant SNPs, the potential candidate genes were predicted within ±200 kb of the borders of the significant SNPs. Pairwise LD was determined by calculation of *r*^2^ (the square of the correlation coefficient between SNP states) using PLINK. LD blocks were analyzed using LDheatmap version 0.99 (Shin et al., 2006), and candidate LD blocks were estimated using *r*^2^ > 0.8. The transcriptomic data of rice under THMM stress were extracted from the supplemental data of the related references and NCBI GEO database (Huang et al., 2012; Yu et al., 2012; Ogo et al., 2014). The expression profile of the candidate genes was analyzed using the Bio-Analytic Resource Plant Biology database^1^. The differences in THMM concentration between the haplotypes were statistically analyzed using Welch’s *t*-test (two haplotypes) or Tukey’s test (more than two haplotypes). All SNP variations across genes were extracted from the whole-genome resequencing data of 3 000 Rice Genomes Project (3 K filtered dataset) (Mansueto et al., 2017; Wang et al., 2018).

## Results

### Grain THMM Concentrations in Rice Grown in Contaminated Farmlands

**Figure 1:**
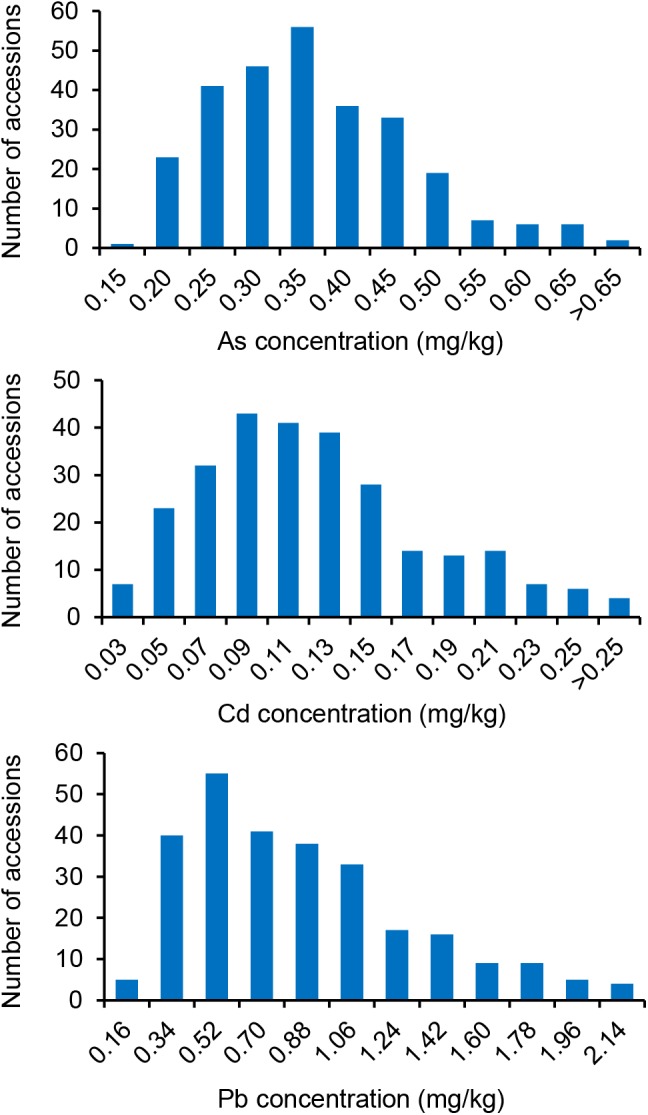
Diversity of grain toxic heavy metal and metalloid concentrations of the 276 global rice accessions grown at the multi-contaminated farmland. Histograms of grain (brown rice) As, Cd, and Pb concentrations (averages of 2 years) are shown separately.

Significant differences in the concentration of the three THMMs (As, Cd, and Pb) in grain (brown rice) were noted among the 276 global representative rice accessions (Supplementary Table S1) grown in a multi-contaminated farm with high soil As, Cd, and Pb levels, suggesting vast variations of grain THMM accumulation abilities of rice (Figure 1 and Table 1). The means for grain As, Cd, and Pb concentrations were 0.34 ± 0.11, 0.12 ± 0.06, and 0.78 ± 0.45 mg/kg, respectively (Table 1). The grain As concentration showed minimum variation with a 6.25-fold difference, whereas grain Cd concentration showed maximum variation with a 23.57-fold difference (Table 1). The grain As and Pb concentrations of most accessions were over the maximum permitted level for grain commercialization, but the grain Cd concentration of most accessions were below the permitted level (Figure 1). Flooded condition during cultivation could be responsible for the relatively low accumulation of Cd in grain in our study. The accessions with low grain THMM accumulation are listed in Supplementary Table S4 for rice breeding.

**Table 1:** Summary of toxic heavy metal and metalloid concentrations in rice grain (brown rice) of the 276 accessions (mg/kg).

Metal	Mean	*SD*^a^	CV^b^	Minimum	Maximum	Ratio^c^	Skewness	Kurtosis	Heritability^d^
As	0.34	0.11	0.32	0.12	0.75	6.25	0.74	0.52	0.73
Cd	0.12	0.06	0.50	0.01	0.33	23.57	0.80	0.65	0.35
Pb	0.78	0.45	0.58	0.11	2.08	18.91	0.88	0.16	0.21

^a^*SD, standard deviation. ^*c*^CV, coefficient of variation. ^*b*^Ratio, maximum/minimum ratio. ^*d*^Broad sense heritability (H^*2*^)*.

Compared to the normal distribution, the frequency distributions of all three grain THMM concentrations, especially that for Pb, showed unique stronger peaks and longer right tails (Figure 1 and Table 1). The broad-sense heritability (*H*^2^) of grain As, Cd, and Pb concentrations were 0.73, 0.35, and 0.21, respectively, suggesting that environmental factors have an important impact on grain THMM accumulation, especially for Cd and Pb (Table 1). Correlation tests showed a significant correlation between each pair of 2-year repeats for grain THMM concentrations (all *P* ≤ 4.52 × 10^-4^, Supplementary Table S2), enabling the examination of the genetic basis for grain THMM concentrations. Two-way *ANOVA* was then performed to examine both the genotype effect and year effect on grain THMM concentrations. Significant genotype effects were observed (all *P* ≤ 2.37 × 10^-4^, Supplementary Table S3), while no significant year effect was found for all three grain THMM concentrations (all *P* ≥ 0.41, Supplementary Table S3). These results together suggest that the environmental factors affecting grain THMM concentrations are complex and remain unclear. Correlation analysis performed among the three grain THMM concentrations revealed a significant positive correlation between grain Cd and Pb concentrations (*r* = 0.63, *P* = 4.87 × 10^-34^) and a negative correlation between grain As and Cd concentrations (*r* = -0.15, *P* = 0.02). This suggests that grain Cd accumulation could be impacted by similar factors as those for grain Pb accumulation, but different from those for grain As accumulation. No significant correlation was noted between grain As and Pb concentrations.

### Variation of Grain THMM Concentrations Among Different Rice Subpopulations

Previous PC analysis revealed a clear population structure of the rice diversity panel (Zhao et al., 2011). We extracted 415,754 high-quality SNPs (missing rate, <0.2; minor allele frequency, >0.05) from three SNP arrays for the selected 276 accessions, with a high density of 0.90 kb/marker (Table 2). The PC analysis based on these 416 K SNPs also indicated the same population structure for the selected accessions (Supplementary Figure S1). Next, we performed correlation analysis between grain THMM concentrations and the top four PCs. We found a positive correlation between PC3 and all three grain THMM concentrations and a negative correlation between PC4 and grain As concentration, suggesting that genetic diversity plays a role in grain THMM accumulation abilities (Table 3).

**Table 2:** Distributions of single nucleotide polymorphism markers on rice chromosomes.

Chromosome	SNP number	Length (Mb)	Density (kb)
Chr01	50,834	43.2	0.85
Chr02	40,558	35.9	0.89
Chr03	39,310	36.3	0.92
Chr04	36,236	35.5	0.98
Chr05	30,847	29.7	0.96
Chr06	34,289	31.1	0.91
Chr07	30,994	29.7	0.96
Chr08	32,353	28.4	0.88
Chr09	26,000	22.9	0.88
Chr10	27,128	23.1	0.85
Chr11	36,908	29.0	0.79
Chr12	30,297	27.4	0.90
Total	415,754	372.2	0.90

**Table 3:** Correlation analysis between grain toxic heavy metal and metalloid concentrations and top four principal components (PCs) for genetic variations of the rice accessions.

	As	Cd	Pb
PC1	-0.009	-0.115	0.003
PC2	-0.035	0.060	0.058
PC3	0.295***	0.178**	0.238***
PC4	-0.161**	0.078	-0.082

*^∗∗^P < 0.01, ^∗∗∗^P < 0.001*.

**Figure 2:**
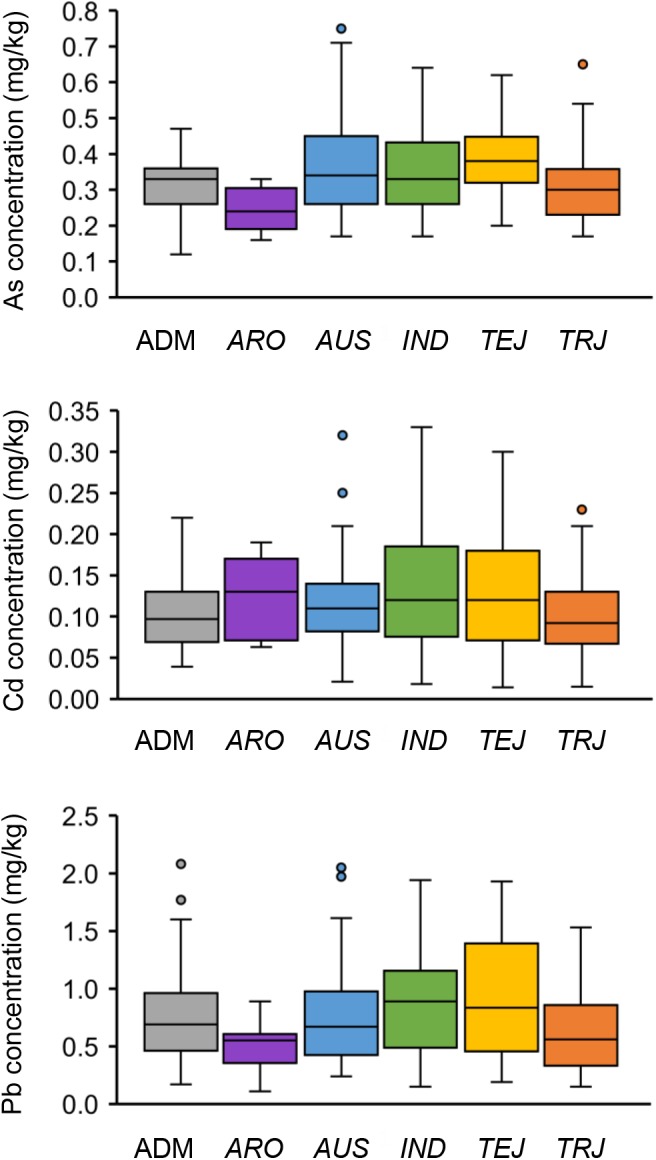
Performance of grain toxic heavy metal and metalloid concentrations of different rice subpopulations. Boxplots of grain As, Cd, and Pb concentrations (averages of 2 years) are shown separately. ADM, admixed; *ARO*, *aromatic*; *AUS*, *aus*; *IND*, *indica*; *TEJ*, *temperate japonica*; *TRJ*, *tropical japonica*.

Population structure analysis of these 276 accessions determined the optimum subpopulation number to be five (McCouch et al., 2016). The population consisted of five distinct subpopulations [*aromatic* (*ARO*), *aus* (*AUS*), *indica* (*IND*), *temperate japonica* (*TEJ*), and *tropical japonica* (*TRJ*)] and an admixed (ADM) subpopulation (Supplementary Table S1; Zhao et al., 2011). The top four PCs clearly separated these subpopulations except the ADM subpopulation (Supplementary Figure S1). Different subpopulations showed variations in grain THMM concentrations (Figure 2). The *ARO* subpopulation showed the lowest grain As and Pb concentration, and the *TRJ* subpopulation had the lowest grain Cd concentration; *TEJ*, *ARO*, and *IND* subpopulations had the highest grain As, Cd, and Pb concentrations, respectively (Figure 2). These data indicate a strong relationship of population structure with grain THMM concentrations. Further, we noted remarkable variations in grain THMM concentrations within some subpopulations, for example, *AUS*, *IND*, and *TEJ* subpopulations showed large variations of grain As, Cd, and Pb concentrations, respectively (Figure 2), suggesting the existence of major QTLs within the rice subpopulations.

### Genome-Wide Association Mapping in the Rice Population

**Figure 3:**
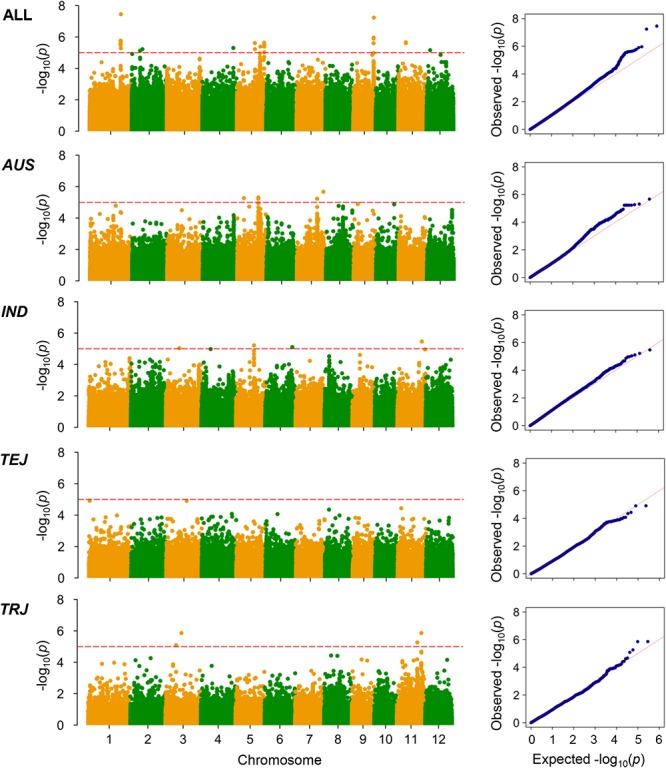
Genome-wide association mapping results for grain As concentrations. Manhattan plots of association mapping results (*P*-values) are shown at the **(left)**, and the corresponding Quantile–Quantile plots are shown at the **(right)**. Association mappings were performed for the entire population with all 276 accessions, as well as for each subpopulation with adequate accessions (*AUS*, *aus*; *IND*, *indica*; *TEJ*, *temperate japonica*; *TRJ*, *tropical japonica*). The red dashed lines indicate the threshold for significance (log_10_-transformed *P*-values, <5.0) according to a previous study (McCouch et al., 2016).

**Figure 4:**
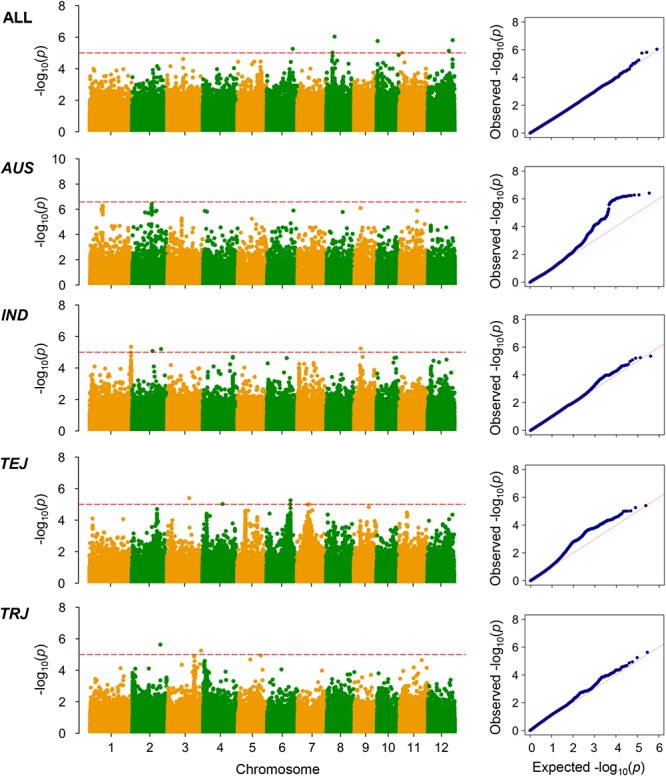
Genome-wide association mapping results for grain Cd concentrations. Manhattan plots of association mapping results (*P*-values) are shown at the **(left)**, and the corresponding Quantile–Quantile plots are shown at the **(right)**. Association mappings were performed for the entire population with all 276 accessions, as well as for each subpopulation with adequate accessions (*AUS*, *aus*; *IND*, *indica*; *TEJ*, *temperate japonica*; *TRJ*, *tropical japonica*). The red dashed lines indicate the threshold for significance (log_10_-transformed *P*-values, <5.0 or 6.6 for *AUS* subpopulation).

**Figure 5:**
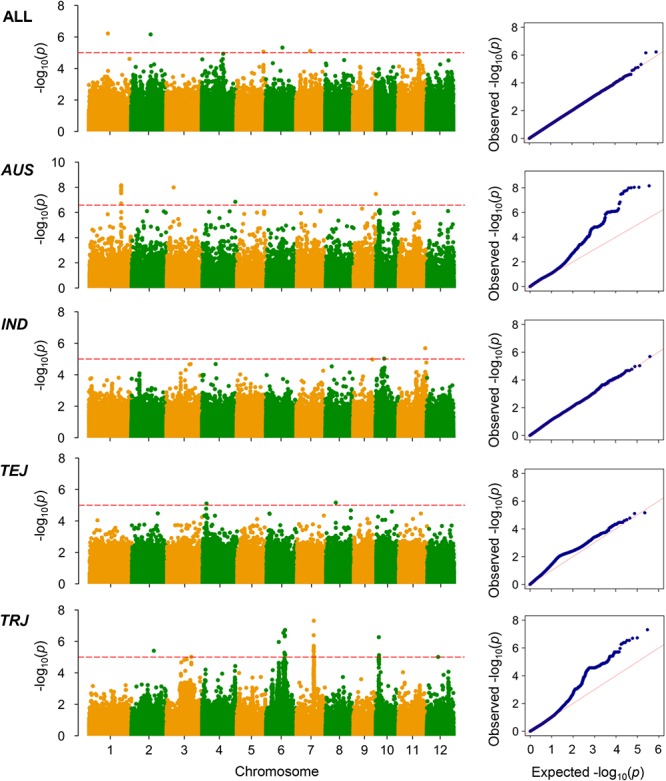
Genome-wide association mapping results for grain Pb concentrations. Manhattan plots of association mapping results (*P*-values) are shown at the **(left)**, and the corresponding Quantile–Quantile plots are shown at the **(right)**. Association mappings were performed for the entire population with all 276 accessions, as well as for each subpopulation with adequate accessions (*AUS*, *aus*; *IND*, *indica*; *TEJ*, *temperate japonica*; *TRJ*, *tropical japonica*). The red dashed lines indicate the threshold for significance (log_10_-transformed *P*-values < 5.0 or 6.6 for *AUS* subpopulation).

**Figure 6:**
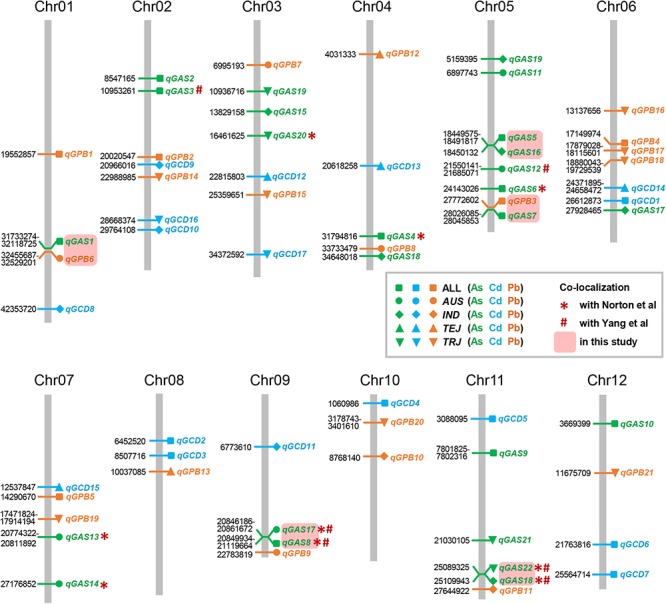
Chromosomal distribution of the quantitative trait loci (QTLs) detected in this study and comparison with previously identified QTLs. The physical location (Mb) of each peak SNP is indicated on the left side of each column, which represents a chromosome. The corresponding QTL is indicated on the right of the column. *AUS*, *aus*; *IND*, *indica*; *TEJ*, *temperate japonica*; *TRJ*, *tropical japonica*.

The LD decay analysis yielded similar results as those reported previously (Supplementary Figure S2) (McCouch et al., 2016). We used linear mixed model for genome-wide association mapping, which has been widely used and validated to be efficient (Yang et al., 2014). The impact of population structure in association mapping was determined by estimating the genetic relatedness. The association mapping performed using 416 K high-quality SNPs identified a total of 22 QTLs (21 non-redundant loci) reaching the thresholds (*P* < 1.00 × 10^-5^; Figure 3–6 and Tables 4–6). The detection power was the highest for grain As concentration, and 10 QTLs were detected in the rice population (Figure 3 and Table 4). Seven QTLs were found to be responsible for grain Cd concentration, and the remaining five were responsible for grain Pb concentration (Figure 4, 5 and Tables 5, 6). A QTL in chromosome 5 was detected for both grain As and Pb concentrations (*qGAS7* and *qGPB3*; Figure 3, 5, 6 and Tables 4, 6).

**Table 4:** Summary of quantitative trait loci identified for grain As concentrations in rice.

Locus	Dataset	Chr.^a^	SSN^b^	Peak SNP^c^	Location	Allele^d^	MAF^e^	Effect^f^	*P*-value
*qGAS1*	ALL	1	8	SNP-1.32110595	32111640	T/G	0.34	0.06	3.55 × 10^-8^
*qGAS2*	ALL	2	1	SNP-2.8547163	8547165	T/A	0.08	0.07	7.53 × 10^-6^
*qGAS3*	ALL	2	1	SNP-2.10953257	10953261	A/G	0.07	0.07	6.02 × 10^-6^
*qGAS4*	ALL	4	1	SNP-4.31609708	31794816	T/C	0.07	0.07	4.98 × 10^-6^
*qGAS5*	ALL	5	2	id5007352	18449575	A/G	0.09	0.08	2.43 × 10^-6^
*qGAS6*	ALL	5	1	SNP-5.24080444	24143026	C/T	0.10	0.06	4.14 × 10^-6^
*qGAS7*	ALL	5	4	SNP-5.27975384	28038030	A/G	0.06	0.09	2.34 × 10^-6^
*qGAS8*	ALL	9	4	SNP-9.21119182	21119664	T/C	0.21	0.06	5.88 × 10^-8^
*qGAS9*	ALL	11	2	SNP-11.7798060	7802316	A/G	0.06	0.08	2.20 × 10^-6^
*qGAS10*	ALL	12	1	SNP-12.3668397	3669399	G/A	0.06	0.08	6.96 × 10^-6^
*qGAS11*	*AUS*	5	1	SNP-5.6897684	6897743	A/T	0.13	0.13	5.41 × 10^-6^
*qGAS12*	*AUS*	5	2	SNP-5.21487581	21550141	T/C	0.30	0.10	4.89 × 10^-6^
*qGAS13*	*AUS*	7	4	SNP-7.20773328	20774322	A/G	0.15	0.15	5.92 × 10^-6^
*qGAS14*	*AUS*	7	1	SNP-7.27175857	27176852	C/T	0.29	0.09	2.15 × 10^-6^
*qGAS15*	*IND*	3	1	SNP-3.13827874	13829158	T/C	0.23	0.07	9.12 × 10^-6^
*qGAS16*	*IND*	5	1	SNP-5.18389617	18450132	G/A	0.26	-0.07	6.03 × 10^-6^
*qGAS17*	*IND*	6	1	SNP-6.27927466	27928465	T/G	0.13	0.08	7.82 × 10^-6^
*qGAS18*	*IND*	11	1	SNP-11.24643782	25109943	C/T	0.24	0.07	3.43 × 10^-6^
*qGAS19*	*TRJ*	3	1	SNP-3.10935433	10936716	G/A	0.08	0.09	8.26 × 10^-6^
*qGAS20*	*TRJ*	3	1	SNP-3.16460269	16461625	C/T	0.08	0.10	1.38 × 10^-6^
*qGAS21*	*TRJ*	11	1	id11007895	21030105	C/T	0.06	0.10	5.51 × 10^-6^
*qGAS22*	*TRJ*	11	1	SNP-11.24623164	25089325	C/T	0.08	0.10	1.39 × 10^-6^

*Threshold for significance, P-value < 1.00 × 10^-*5*^ (<2.62 × 10^-*7*^ for AUS subpopulation). ^*a*^Chr., chromosome. ^*b*^SSN, significant SNP number. ^*c*^SNP with the best P value. ^*d*^Allele, minor/major alleles. ^*e*^MAF, minor allele frequency. ^*f*^Effect, allele effect with respect to the minor allele*.

**Table 5:** Summary of quantitative trait loci identified for grain Cd concentrations in rice.

Locus	Dataset	Chr.^a^	SSN^b^	Peak SNP^c^	Location	Allele^d^	MAF^e^	Effect^f^	*P*-value
*qGCD1*	ALL	6	1	SNP-6.26611875	26612873	T/C	0.12	-0.03	5.50 × 10^-6^
*qGCD2*	ALL	8	1	SNP-8.6451524	6452520	A/T	0.23	0.03	9.55 × 10^-6^
*qGCD3*	ALL	8	1	SNP-8.8506719	8507716	C/T	0.14	0.02	9.14 × 10^-7^
*qGCD4*	ALL	10	1	SNP-10.1059961	1060986	G/T	0.17	-0.02	1.74 × 10^-6^
*qGCD5*	ALL	11	1	SNP-11.3083997	3088095	C/A	0.12	-0.02	9.80 × 10^-6^
*qGCD6*	ALL	12	1	SNP-12.21730362	21763816	A/T	0.06	0.04	7.41 × 10^-6^
*qGCD7*	ALL	12	1	SNP-12.25531108	25564714	T/C	0.07	-0.03	1.54 × 10^-6^
*qGCD8*	*IND*	1	1	SNP-1.42352675	42353720	A/G	0.26	0.05	4.52 × 10^-6^
*qGCD9*	*IND*	2	1	SNP-2.20960147	20966016	A/G	0.09	0.07	8.34 × 10^-6^
*qGCD10*	*IND*	2	1	SNP-2.29758238	29764108	T/C	0.11	0.07	6.27 × 10^-6^
*qGCD11*	*IND*	9	1	SNP-9.6772609	6773610	T/C	0.12	0.06	5.82 × 10^-6^
*qGCD12*	*TEJ*	3	1	SNP-3.22814291	22815803	T/C	0.32	0.04	3.96 × 10^-6^
*qGCD13*	*TEJ*	4	1	SNP-4.20446300	20618258	T/C	0.28	0.04	9.48 × 10^-6^
*qGCD14*	*TEJ*	6	2	SNP-6.24657474	24658472	C/T	0.45	0.04	5.57 × 10^-6^
*qGCD15*	*TEJ*	7	1	SNP-7.12536853	12537847	C/A	0.20	0.05	9.91 × 10^-6^
*qGCD16*	*TRJ*	2	1	SNP-2.28662504	28668374	T/G	0.09	0.04	2.37 × 10^-6^
*qGCD17*	*TRJ*	3	1	SNP-3.34365461	34372592	G/A	0.12	0.04	5.70 × 10^-6^

*Threshold for significance, P-value < 1.00 × 10^-*5*^. ^*a*^Chr., chromosome. ^*b*^SSN, significant SNP number. ^*c*^SNP with the best P-value. ^*d*^Allele, minor/major alleles. ^*e*^MAF, minor allele frequency. ^*f*^Effect, allele effect with respect to the minor allele*.

**Table 6:** Summary of quantitative trait loci identified for grain Pb concentrations in rice.

Locus	Dataset	Chr.^a^	SSN^b^	Peak SNP^c^	Location	Allele^d^	MAF^e^	Effect^f^	*P*-value
*qGPB1*	ALL	1	1	SNP-1.19551811	19552857	A/C	0.12	0.24	6.01 × 10^-7^
*qGPB2*	ALL	2	1	SNP-2.20014678	20020547	A/G	0.08	0.27	6.92 × 10^-7^
*qGPB3*	ALL	5	1	SNP-5.27709958	27772602	A/G	0.18	-0.33	8.57 × 10^-6^
*qGPB4*	ALL	6	1	SNP-6.17148976	17149974	A/G	0.05	0.27	4.75 × 10^-6^
*qGPB5*	ALL	7	1	SNP-7.14289676	14290670	T/C	0.06	0.25	7.75 × 10^-6^
*qGPB6*	*AUS*	1	10	SNP-1.32496771	32497816	T/C	0.10	0.68	6.85 × 10^-9^
*qGPB7*	*AUS*	3	1	SNP-3.6994190	6995193	A/T	0.19	0.51	1.03 × 10^-8^
*qGPB8*	*AUS*	4	1	SNP-4.33548358	33733479	G/A	0.11	0.56	1.42 × 10^-7^
*qGPB9*	*AUS*	9	1	SNP-9.22783337	22783819	T/C	0.08	0.66	3.36 × 10^-8^
*qGPB10*	*IND*	10	1	SNP-10.8697001	8768140	C/T	0.10	0.45	9.45 × 10^-6^
*qGPB11*	*IND*	11	1	SNP-11.27173308ga	27644922	A/G	0.30	0.31	2.07 × 10^-6^
*qGPB12*	*TEJ*	4	1	SNP-4.4026939	4031333	C/T	0.15	0.36	7.82 × 10^-6^
*qGPB13*	*TEJ*	8	1	SNP-8.10036088	10037085	T/C	0.44	-0.30	6.90 × 10^-6^
*qGPB14*	*TRJ*	2	1	SNP-2.22983115	22988985	A/G	0.12	0.29	3.94 × 10^-6^
*qGPB15*	*TRJ*	3	1	SNP-3.25357731	25359651	C/T	0.08	0.35	9.55 × 10^-6^
*qGPB16*	*TRJ*	6	1	SNP-6.13136656	13137656	A/T	0.09	0.35	1.09 × 10^-6^
*qGPB17*	*TRJ*	6	2	SNP-6.17878030	17879028	T/C	0.09	0.37	2.86 × 10^-7^
*qGPB18*	*TRJ*	6	7	SNP-6.19647081	19648079	C/A	0.08	0.40	1.88 × 10^-7^
*qGPB19*	*TRJ*	7	23	SNP-7.17697649	17698643	A/G	0.14	0.32	4.88 × 10^-8^
*qGPB20*	*TRJ*	10	2	SNP-10.3177719	3178743	A/T	0.14	0.32	5.43 × 10^-7^
*qGPB21*	*TRJ*	12	1	SNP-12.11673044	11675709	T/G	0.08	0.35	9.75 × 10^-6^

Threshold for significance, P-value < 1.00 × 10^-*5*^ (<2.62 × 10^-*7*^ for AUS subpopulation). ^*a*^Chr., chromosome. ^*b*^SSN, significant SNP number. ^*c*^SNP with the best P-value. ^*d*^Allele, minor/major alleles. ^*e*^MAF, minor allele frequency. ^*f*^Effect, allele effect with respect to the minor allele.

### Genome-Wide Association Mapping in the Rice Subpopulations

Although the impact of population structure has been considered during association mapping, we still performed association mapping for each of the subpopulations considering a strong relationship between population structure and grain THMM concentrations. The association mapping was not possible for the *ARO* subpopulation because of very limited accessions and was not performed for the ADM subpopulation. For the *AUS*, *IND*, *TEJ*, and *TRJ* subpopulations, 190,616, 198,790, 117,880, and 147,039 high-quality SNPs (missing rate, <0.2; minor allele frequency, >0.05) were successfully extracted, respectively. We detected four QTLs in each of the *AUS*, *IND*, and *TRJ* subpopulations for grain As concentration (totally 12 QTLs; Figure 3 and Table 4). For grain Cd concentration, four, four, and two QTLs were detected in the *IND*, *TEJ*, and *TRJ* subpopulations, respectively (totally 10 QTLs; Figure 4 and Table 5). For grain Pb concentration, four, two, two, and eight QTLs were identified in the *AUS*, *IND*, *TEJ*, and *TRJ* subpopulations, respectively (totally 16 QTLs; Figure 5 and Table 6). Consequently, 8, 10, 6, and 14 QTLs were discovered in the *AUS*, *IND*, *TEJ*, and *TRJ* subpopulations, respectively. Three QTLs (*qGAS16*, *qGAS17*, and *qGPB6*) were co-localized with the QTLs detected in the entire rice population (Figure 6). Additionally, two QTLs for grain As concentration, one in the *AUS* subpopulation (*qGAS18*) and the other in the *TRJ* subpopulation (*qGAS22*), were co-localized on the chromosome 11 (Figure 6).

### Candidate Gene Prediction for Grain THMM Accumulation

We determined whether the identified QTLs harbored known THMM-related genes. *qGAS8* and *qGAS17* contain *OsPIP2;7*, encoding an aquaporin involved in arsenite permeability and transport (Mosa et al., 2012). *qGAS12* contains *OsARM1* and *OsPT23*. The former encodes a MYB transcription factor responsible for As root-to-shoot translocation (Wang et al., 2017), and the latter encodes a phosphate transporter involved in the uptake of arsenate (Yu et al., 2012; Clemens and Ma, 2016). Next, we predicted the possible candidate genes in the regions of QTLs (±200 kb of the significant SNPs; Supplementary Table S5). Genes encoding proteins with transport functions are known to play important roles in THMM accumulation in plants (Clemens and Ma, 2016). We found that 61.8% of the identified QTLs contained genes encoding transporter-related proteins (Supplementary Table S5). Notably, high concentrations of THMMs in the soil used in this study could induce toxic effect and stress for rice plants. Interestingly, *OsPIP2;7* and *OsARM1* harbored in the identified QTLs (*qGAS8*, *qGAS12*, and *qGAS17*) for grain As accumulation were reported to be involved in As tolerance and detoxification (Mosa et al., 2012; Wang et al., 2017). By examining the THMM stress-responsive genes previously identified using transcriptome analysis in rice (Huang et al., 2012; Yu et al., 2012; Ogo et al., 2014), we found that 12 and six QTLs for grain As and Cd concentrations contain genes responding to As and Cd stress, respectively, including *OsARM1* (Supplementary Table S6).

**Figure 7:**
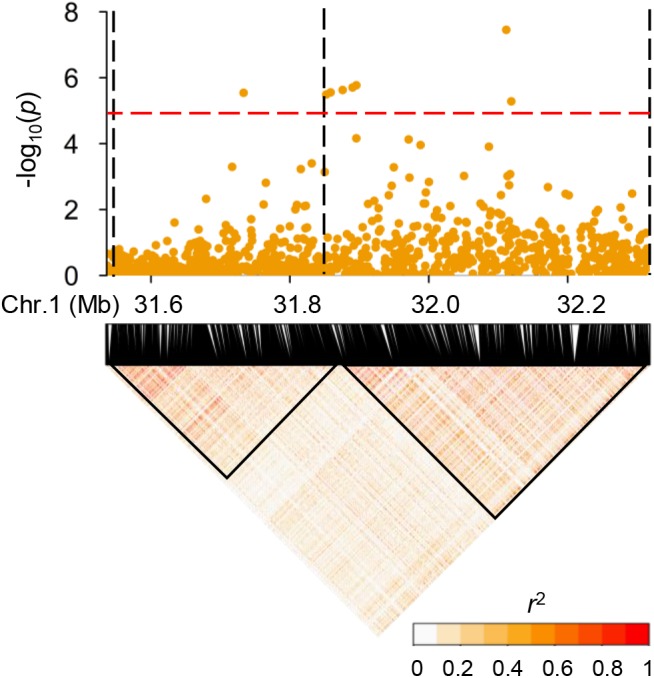
Linkage disequilibrium (LD) blocks at the region of *qGAS1*. The **(upper)** panel shows the local Manhattan plot of association mapping results (*P*-values) at the region of *qGAS1*. The red dashed line indicates the threshold for significance (log_10_-transformed *P*-value, <5.0). The black dashed lines indicate the borders of LD blocks. The **(lower)** panel shows local LD heatmap of the region of *qGAS1*. The LD blocks are indicated by a black triangle.

**Figure 8:**
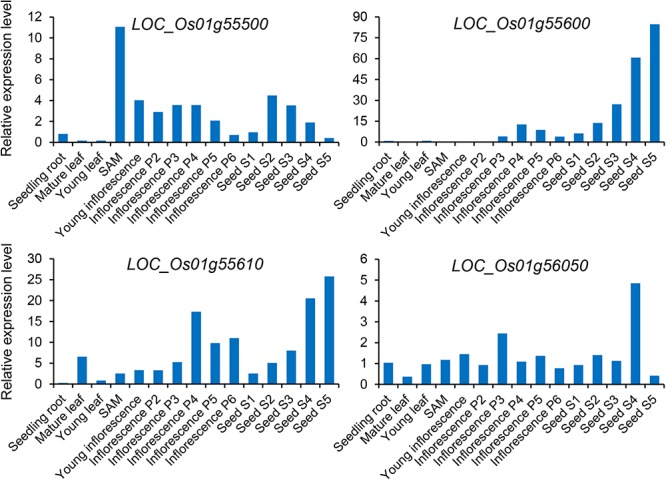
Expression profiles of four potential candidate genes for the *qGAS1* locus. The relative expression level indicates the fold change of the signal for the gene relative to the control signal. The different stages of panicle and seed development are based on the study of Itoh et al. (2005). SAM, shoot apical meristem.

**Figure 9:**
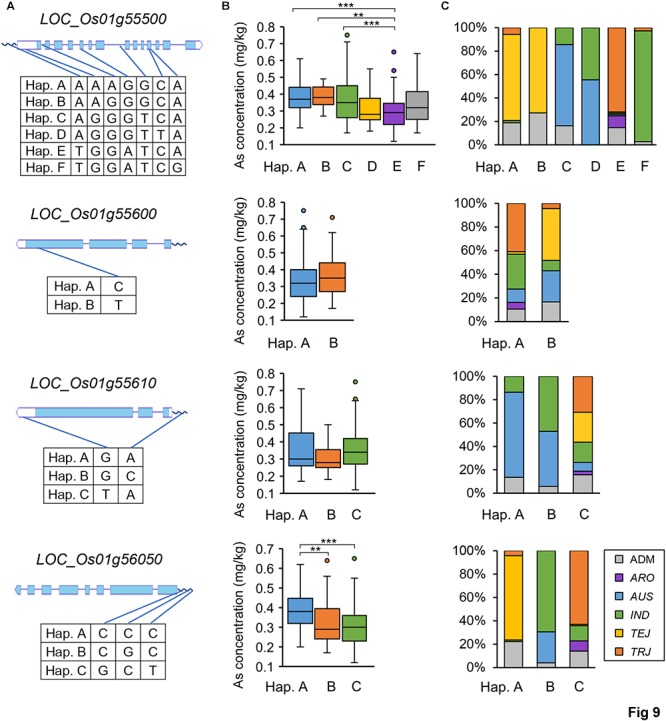
Haplotype analyses of four potential candidate genes for the *qGAS1* locus. **(A)** The haplotypes of each gene based on the SNPs used in this study. The SNP distributions in the gene structures (wave lines for promoters) are indicated by blue lines. **(B)** Boxplots for grain As concentrations of different haplotypes. ^∗∗^*P* < 0.01, ^∗∗∗^*P* < 0.001. **(C)** Haplotype distributions in the rice population. The 100% stacked columns indicate the percentages of respective subpopulations for each haplotype, and different subpopulations are marked by different colors. Hap., haplotype.

Subsequently, we focused on *qGAS1*, which had the best *P*-value among the QTLs detected in the entire population. Analysis of LD blocks revealed two blocks in the region of *qGAS1* (Figure 7). Almost all significant SNPs were located in the second block (Figure 7). We found four transporter-related genes in this block. According to protein homology, *LOC_Os01g55500* encodes a nucleobase–ascorbate transporter, *LOC_Os01g55600* and *LOC_Os01g55610* encode nitrate transporters, and *LOC_Os01g56050* encodes a MATE (also known as multi-antimicrobial extrusion) transport protein. The expression profiles of the four genes showed that they were mainly expressed in seeds/inflorescences, except *LOC_Os01g55500* (which was mainly expressed in the shoot apical meristem; Figure 8). Next, we performed haplotype analysis for these genes (Figure 9A). *LOC_Os01g55500* and *LOC_Os01g56050* showed different grain As concentrations between some of the haplotypes (Figure 9B). Taken together, our findings suggest that *LOC_Os01g56050* might be a possible candidate gene for *qGAS1.*

In our SNP dataset, only three SNP markers were found on the promoter (within 1 kb) of *LOC_Os01g56050*, which divided the gene alleles into three haplotypes (Figure 9A). Haplotype A with the highest grain As concentration was primarily distributed in the *TEJ* subpopulation, whereas haplotype C with the lowest grain As concentration was primarily distributed in the *TRJ* subpopulation (Figure 9B,C). We then extracted all the SNPs on *LOC_Os01g56050* from the whole-genome resequencing data of rice population (Wang et al., 2018). The eight SNPs found on this gene also divided the gene alleles into three major haplotypes (Supplementary Table S7). Two of the SNPs caused non-synonymous changes between the haplotypes (valine^213^ to isoleucine^213^ and lysine^459^ to isoleucine^459^; Supplementary Table S7). The other SNPs were located in the introns or caused synonymous changes (Supplementary Table S7). Both valine and isoleucine are hydrophobic amino acids and have similar biochemical characters, whereas lysine and isoleucine have distinct biochemical characters (lysine is a basic amino acid). Furthermore, lysine^459^ is a relatively conserved residue in the MATE domain (Supplementary Figure S3). Therefore, the difference of lysine^459^ (haplotypes A and B) and isoleucine^459^ (haplotypes C) could have an impact on the protein activity of *LOC_Os01g56050* alleles.

## Discussion

Although the genetic analysis of grain THMM accumulation is important, the association mapping of the causal genetic loci has not yet been comprehensively conducted in rice grown in heavily multi-contaminated farmlands. We completed the association mapping of three THMM concentrations in rice grain by using 276 accessions and 416 K SNP markers. In addition to the 22 QTLs (21 non-redundant loci) identified in the entire population, we discovered 48 QTLs (47 non-redundant loci) in the subpopulations. In all, we detected 60 QTLs (55 non-redundant loci) for grain THMM concentrations in rice. The genes involved in As accumulation have been investigated widely, but only three of 22 QTLs for grain As concentration contain known As-related genes. No known Cd-/Pb-related genes were found in the 17/21 QTLs for grain Cd/Pb concentration. These findings suggest that the genetic regulation of grain THMM accumulation is very complex and remains largely unknown.

Notably, the detection powers for grain Cd and Pb concentrations in the entire population were lower than those for grain As concentration (Figure 3 and Table 4). This could be because the heritability of As accumulation is considerably higher than that of Cd (flooded condition) and Pb accumulation (Table 1), as revealed by previous studies (Norton et al., 2014b; Pinson et al., 2015). We observed a significant positive correlation between grain Cd and Pb concentrations, but failed to detect some common QTLs governing both their concentrations. This implies that both grain Cd and Pb accumulation are impacted by similar non-genetic factors. Cd and Pb accumulation in plants is known to be strongly affected by environments (Norton et al., 2014b; Pinson et al., 2015; Clemens and Ma, 2016). Therefore, environmental factors, rather than genotypes, might be attributed to this positive correlation. Both the broad-sense heritabilities of grain Cd and Pb concentrations were lower than 40% (Table 1), supporting the large impact of environmental factors on Cd and Pb accumulation in rice grain. Compared with those under unflooded field condition, the average grain concentrations of As were higher and those of Cd were lower under flooded field condition (Pinson et al., 2015). Consistent with this, we found a negative correlation between grain As and Cd concentrations under our flooded field condition, which was also responsible for the relatively low accumulation of grain Cd in the rice accessions grown in the soil with high Cd concentrations (Figure 1 and Table 1). We only found a few QTLs involved in the accumulation of two kinds of THMMs, suggesting that the accumulation of different THMMs in grain is differently regulated.

Because of the relatively small number of accessions, the detection powers for association mapping could have decreased in the respective subpopulations. Unexpectedly, we detected several strong peak signals, such as *qGPB19* for grain Pb concentration in *TRJ* subpopulation, which were not detected in the entire population (Figure 3–6 and Tables 4–6). This suggests the existence of major intra-subpopulation QTLs for grain THMM concentrations, which are not detected in the entire population. Subpopulation-specific QTLs might be determined by the presence/absence or different frequencies of specific alleles in one or more subpopulations. A weak population structure in the subpopulations could also benefit the association mapping (Yano et al., 2016). We did not perform association mapping for each PC, because the subpopulations were well consistent with the PCs (Supplementary Figure S1).

Norton et al. (2014a) performed association mapping of grain As [as well as copper, molybdenum, and zinc (Zn)] concentration by using 37 K SNPs. Nine of the QTLs identified in our study were co-localized with those identified in their study (Figure 6). Therefore, 13 loci for grain As concentration were newly identified in our study. Considerably more SNP markers and different field environments might partly account for these differences in QTLs. The association study of grain Cd and Pb accumulation was not performed by Norton et al. (2014a). We also compared our results with those of Yang et al. (2018), which were obtained in normal farmlands. Six of the QTLs for grain As concentration were co-localized with those identified in their study (Figure 6). Surprisingly, none of the QTLs we identified for grain Cd and Pb concentration were co-localized with those in their study. This suggests that environmental factors considerably affect the variations of grain Cd and Pb concentrations, or low and high Cd/Pb concentrations could be regulated by different mechanisms. Contrarily, four QTLs (2 non-redundant loci) for grain As concentration were detected (co-localization) in all three studies (Figure 6). These commonly detected QTLs should be useful for breeding rice varieties with low accumulation of THMMs.

High soil THMM concentrations could impose THMM stress on rice plants and induce stress responses in this study. Some of the QTLs identified for grain THMM accumulation might also be related to THMM tolerance. In line with this assumption, two candidate genes (*OsPIP2;7* and *OsARM1*) in the QTLs for grain As accumulation have been reported to function in As tolerance and detoxification (Mosa et al., 2012; Wang et al., 2017), and 18 QTLs for grain As and Cd concentrations contain genes responding to As or Cd stress. In this study, the soil also contained a high level of Zn (about 500 mg/kg) (Wang et al., 2008; Wu et al., 2013). This could trigger plant responses to the toxic effects, but whether high Zn level directly or indirectly influences THMM accumulation in grain is not yet known. The unclear complex effects of multiplex soil contamination on plants limit the strength of this research. However, multiple contaminations widely exist in farmland near mines and E-waste recycling areas. In the future, the possible multiplex toxic effects of multiple THMMs in soil and the possible interaction effects between THMM accumulation in plants need to be investigated.

The *qGAS1* with the best *P*-value in the entire population was also not detected by Norton et al. (2014a). We examined whether transporter-related genes could be the candidate gene for *qGAS1* (Figure 7–9). *LOC_Os01g56050* encodes a transport protein belonging to the MATE family, which is involved in multiple functions and processes, including metal detoxification (Omote et al., 2006; Takanashi et al., 2014). Several plant MATE transporters have been reported to mediate metal (aluminum, iron, and Cd) translocation and tolerance (Li et al., 2002; Rogers and Guerinot, 2002; Magalhaes et al., 2007; Yokosho et al., 2009, 2011; Maron et al., 2010). The substrates of MATE transporters are usually organic cations (Omote et al., 2006). The MATE transporter encoded by *LOC_Os01g56050* might transport As-ligand complexes such as phytochelatins to decrease As accumulation in grain. The confirmation of *LOC_Os01g56050* as the causative gene for *qGAS1* needs to be further confirmed using CRISPR/Cas9 editing or transgene lines.

We identified several rice accessions with low grain THMM concentrations, which can be used for rice breeding (Supplementary Table S4). However, few accessions showed low accumulation of all three THMMs in grain, suggesting the need of pyramiding breeding for low grain THMM accumulation rice, i.e., introduction of respective loci for low accumulation of different THMMs into one accession. For these accessions with low grain THMM concentrations, we didn’t find a common geographical origin or a clear phylogenetic cluster, suggesting the complex regulation of THMM accumulation. In conclusion, our study reveals the complex regulation of THMMs in rice grain and novel loci and genes governing THMM accumulation. Both genetic and environmental factors contribute to the variation of grain THMM concentrations. The accumulation of different THMMs in grain is regulated by different genetic loci/genes involved in different pathways. Our results can help reduce THMM contents in rice grains and promote marker-assisted breeding of rice with low THMM content.

## Author Contributions

ST and XL designed the research. XL, MC, YP, XS, and PQ performed the experiments. SC, GZ, and ST analyzed the data. ST and XX conceived and supervised the project. SC and ST drafted and revised the manuscript.

## Conflict of Interest Statement

The authors declare that the research was conducted in the absence of any commercial or financial relationships that could be construed as a potential conflict of interest.

## Footnotes

1 http://bar.utoronto.ca

## Abbreviations

LD: linkage disequilibrium
MATE: multidrug and toxin extrusion
PC: principle component
QTL: quantitative trait locus
SNP: single nucleotide polymorphism
THMM: toxic heavy metal and metalloid
