# Supplementary material for: Association Study Reveals Genetic Loci Responsible for Arsenic, Cadmium and Lead Accumulation in Rice Grain in Contaminated Farmlands
Source: Front Plant Sci. 2019 Feb 5;10:61. doi: 10.3389/fpls.2019.00061 (PMC6370710; doi:10.3389/fpls.2019.00061)
Supplement: Supplementary file 8 [file Data_Sheet_1.PDF]

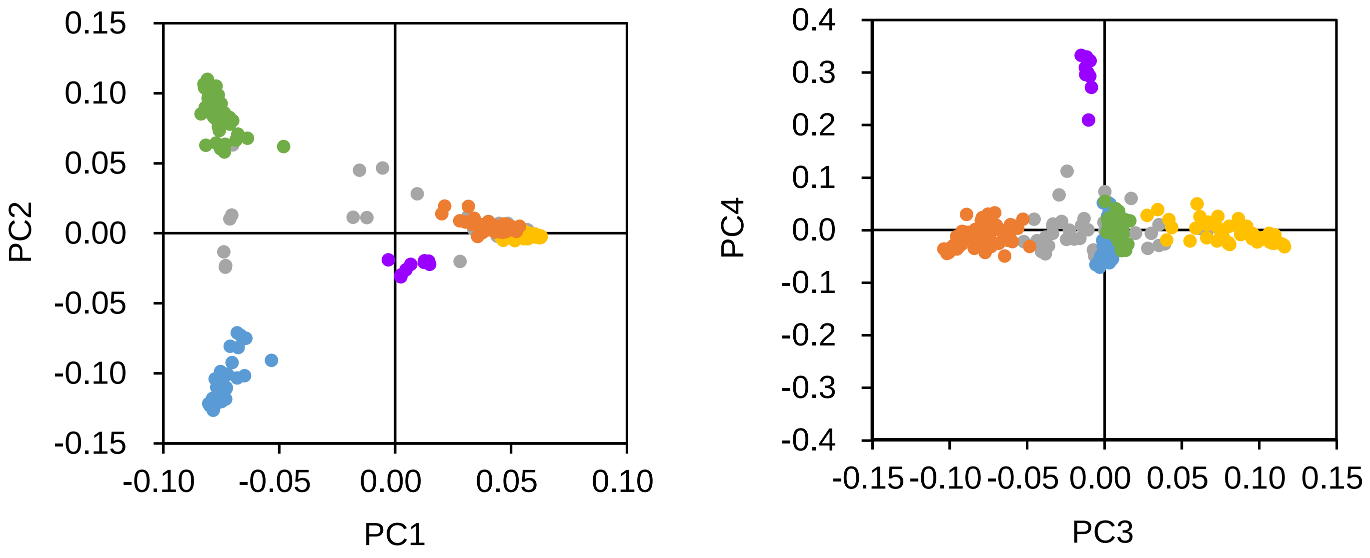

Figure S1. Principal component analysis for the 276 global rice accessions based on 416 K high-quality SNPs. PC1–4 indicate scores of the top four principal components. Rice subpopuplations are marked by different colors (purple, *aromatic*; blue, *aus*; green, *indica*; yellow, *temperate japonica*; orange, *tropical japonica*; grey, admixed).
