# Supplementary material for: Association Study Reveals Genetic Loci Responsible for Arsenic, Cadmium and Lead Accumulation in Rice Grain in Contaminated Farmlands
Source: Front Plant Sci. 2019 Feb 5;10:61. doi: 10.3389/fpls.2019.00061 (PMC6370710; doi:10.3389/fpls.2019.00061)
Supplement: Supplementary file 9 [file Data_Sheet_2.PDF]

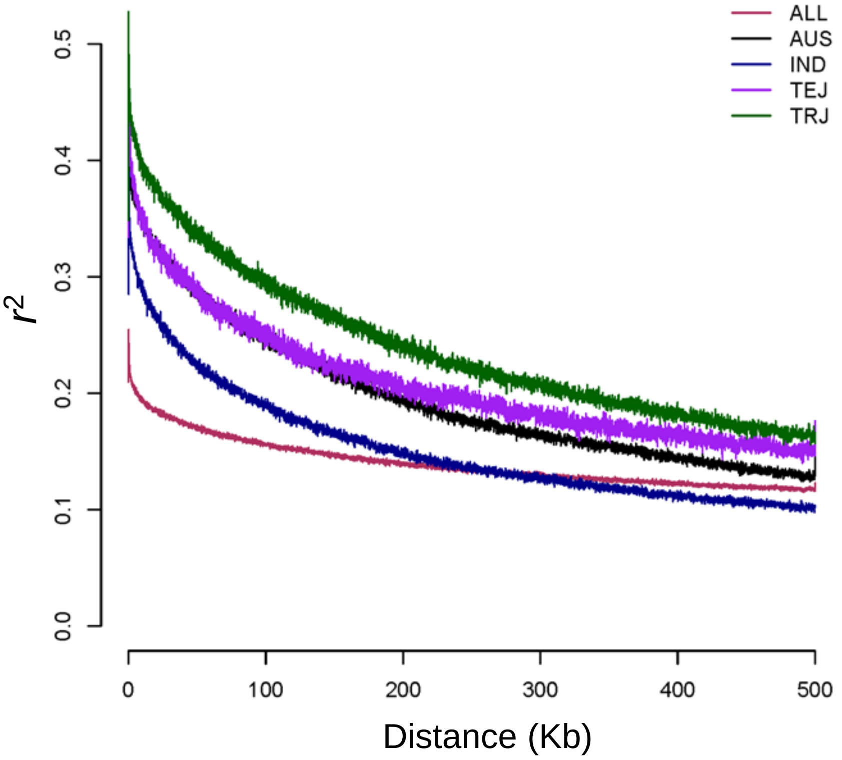

Figure S2. Linkage disequilibrium (LD) decay analysis of the rice population and subpopulations used in this study. LD is used as a function of distance between SNPs.
