# Supplementary material for: Association Study Reveals Genetic Loci Responsible for Arsenic, Cadmium and Lead Accumulation in Rice Grain in Contaminated Farmlands
Source: Front Plant Sci. 2019 Feb 5;10:61. doi: 10.3389/fpls.2019.00061 (PMC6370710; doi:10.3389/fpls.2019.00061)
Supplement: Supplementary file 10 [file Data_Sheet_3.PDF]

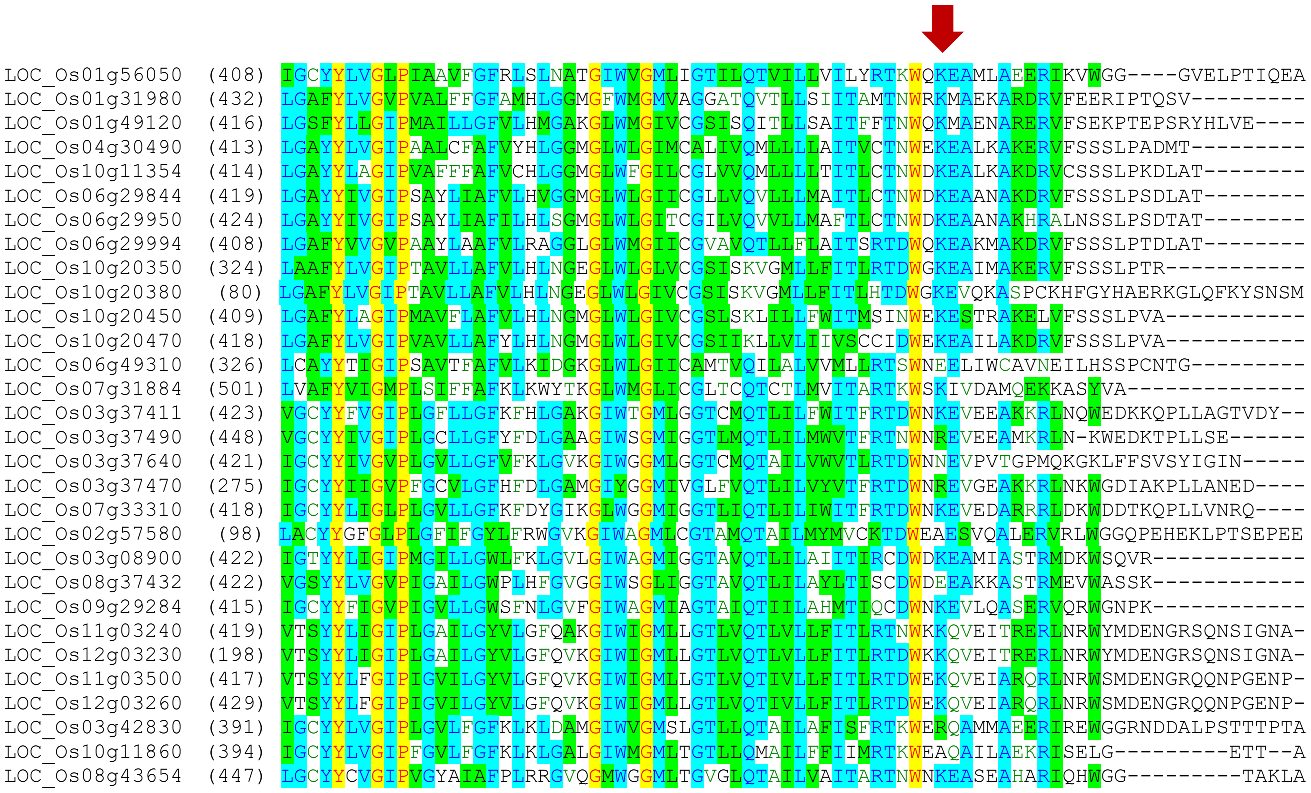

Figure S3. Local protein sequence alignment for MATE proteins in rice. The red arrow points the position of lysine^{459} of the protein encoding by *LOC_Os01g56050*.
